# Hypoxic Melanoma Cells Deliver microRNAs to Dendritic Cells and Cytotoxic T Lymphocytes through Connexin-43 Channels

**DOI:** 10.3390/ijms21207567

**Published:** 2020-10-13

**Authors:** Andrés Tittarelli, Mariela Navarrete, Marcelo Lizana, Francisca Hofmann-Vega, Flavio Salazar-Onfray

**Affiliations:** 1 Programa Institucional de Fomento a la Investigación, Desarrollo e Innovación (PIDi), Universidad Tecnológica Metropolitana (UTEM), Santiago 8940577, Chile; 2 Disciplinary Program of Immunology, Institute of Biomedical Sciences, Faculty of Medicine, Universidad de Chile, Santiago 8380453, Chile; mariela.navarrete.s@gmail.com (M.N.); marcelo.lizana@ug.uchile.cl (M.L.); fcah.vega@gmail.com (F.H.-V.); fsalazar@u.uchile.cl (F.S.-O.); 3 Millennium Institute on Immunology and Immunotherapy, Faculty of Medicine, Universidad de Chile, Santiago 8380453, Chile

**Keywords:** connexin-43, gap junctions, microRNAs, melanoma, hypoxia, cytotoxic T lymphocytes, dendritic cells, miR-192, zeb2

## Abstract

Alterations in microRNA (miRNA) profiles, induced by tumor microenvironment stressors, like hypoxia, allow cancer cells to acquire immune-resistance phenotypes. Indeed, hypoxia-induced miRNAs have been implicated in cancer progression through numerous cancer cell non-autonomous mechanisms, including the direct transfer of hypoxia-responsive miRNA from cancer to immune cells via extracellular vesicles. Connexin-43 (Cx43)-constituted gap junctions (GJs) have also been involved in miRNA intercellular mobilization, in other biological processes. In this report, we aimed to evaluate the involvement of Cx43-GJs in the shift of miRNAs induced by hypoxia, from hypoxic melanoma cells to dendritic cells and melanoma-specific cytotoxic T lymphocytes (CTLs). Using qRT-PCR arrays, we identified that miR-192-5p was strongly induced in hypoxic melanoma cells. Immune cells acquired this miRNA after co-culture with hypoxic melanoma cells. The transfer of miR-192-5p was inhibited when hypoxic melanoma cells expressed a dominant negative Cx43 mutant or when Cx43 expression was silenced using specific short-hairpin RNAs. Interestingly, miR-192-5p levels on CTLs after co-culture with hypoxic melanoma cells were inversely correlated with the cytotoxic activity of T cells and with ZEB2 mRNA expression, a validated immune-related target of miR-192-5p, which is also observed in vivo. Altogether, our data suggest that hypoxic melanoma cells may suppress CTLs cytotoxic activity by transferring hypoxia-induced miR-192-5p through a Cx43-GJs driven mechanism, constituting a resistance strategy for immunological tumor escape.

## 1. Introduction

Both naturally occurring and immunotherapy-induced tumor elimination relies on the coordinated function of different immune cells and a series of events referred to as the cancer-immunity cycle [1]. Briefly, the antitumor immune response begins at the tumor microenvironment (TME), where dendritic cells (DCs) acquire tumor-associated antigens (TAA) and are activated by the recognition of activator signals arising from stressed or dying cancer cells [2]. After this, DCs migrate to tumor-draining lymph nodes where they induce the differentiation and activation of naïve TAA-specific CD8^+^ and CD4^+^ T cells, via the productive engagement of T cell receptors (TCR) by major histocompatibility class I (pMHC-I) or class II (pMHC-II)-antigen peptide complexes, respectively, in conjunction with the recognition of appropriate signals sensed by T cells during the establishment of an immunological synapse [3]. Finally, effector CD8^+^ cytotoxic T lymphocytes (CTL) traffic to and infiltrate the TME, where they kill target cancer cells through mechanisms elicited by the formation of cytotoxic immunological synapses and polarized release of perforin and granzymes-containing cytotoxic granules [4]. Therefore, regardless of their nature, cancer immunotherapy success mainly relies on the elimination of tumor cells by the activities of DCs and CTLs, which are also strongly associated with good prognosis in human cancer patients [5,6].

Unfortunately, cancer cells may develop numerous immuno-evasive mechanisms that allow them to resist natural or therapy-induced immune attacks. Through these mechanisms, tumor cells are capable of modulating themselves and their surroundings in order to promote their survival, growth and invasion, even under a persistent immune pressure. Indeed, stressors present in the TME, such as chronic hypoxia, play determinant roles in promoting tumor cell plasticity and heterogeneity, which finally leads into the acquisition of immune tolerance and tumor progression [7]. For example, it has been shown that hypoxia increases the expression of the immune checkpoint programmed death-ligand 1 (PD-L1) on melanoma cells and tumor infiltrating immune cells, via the transcriptional activity of hypoxia-inducible factor-1α (HIF-1α), leading to lower levels of CTL activation and tumor elimination [8]. Accordingly, tumor hypoxia has been associated with decreased antitumor immunity and poor clinical responses to programmed death-1 (PD-1) blockade therapy in melanoma patients [9].

MicroRNAs (miRNAs) are 19–22 nucleotide long non-coding RNAs that regulate gene expression at a post-transcriptional level. The profile and levels of miRNAs are frequently altered in cancer cells, affecting tumorigenesis and progression, via the induction of sustained proliferation, resistance to cell death, acquisition of invasive phenotypes, and regulating anti-tumor immune responses [10]. Alterations in miRNA profiles in cancer cells can be a consequence of deregulation of specific signaling pathways and/or alterations of their expression in response to TME factors, such as hypoxia [11]. Some of these hypoxia-responsive miRNAs (HRM) have been implicated in cancer progression through numerous cancer cell autonomous mechanisms, including the acquisition of immune-resistance phenotypes. For example, miR-210, a signature of hypoxia in many cell types and a robust target of HIF-1α, decreases the susceptibility of lung cancer and melanoma cells to CTL-mediated lysis, through the down-regulation of different cancer cell genes [12]. Importantly, cancer cell-expressed HRMs also can modulate immune cells of the TME via non-cell-autonomous mechanisms, including the direct mobilization of HRM in extracellular vesicles (EV) (mainly exosomes) from cancer to immune cells, and the subsequent gene targeting in the recipient cells [13,14]. For instance, it has been shown that hypoxic lung cancer-derived miRNA-containing EV negatively impact the antitumor activities of natural killer (NK) cells and macrophages, by directly targeting CD107a and phosphatase and tensin homolog (PTEN) expression, respectively [15,16]. Therefore, there are numerous evidences indicating that hypoxic tumor cells directly reprogram surrounding immune cells in the TME, via exosome-mediated transfer of HRMs, in order to support cancer progression.

Interestingly, the mobility of miRNAs between cells is not limited only to an exosome-mediated mechanism. Indeed, recent data suggests that miRNAs could be transferred between contacting cells via gap junctions (GJs), and it has been proposed as a novel pathway for intercellular regulation of gene expression [17,18]. GJs are connexin (Cx)-formed channels that through the cell-to-cell shuttling of small molecules regulate a plethora of physiological functions, including immunity [19]. Connexin-43 (Cx43), the main GJ protein expressed in immune cells [19], has been shown by us and other groups, to accumulate at immunological synapses, allowing GJ-mediated intercellular communications (GJIC) between lymphocytes and DCs [20,21,22,23], between cytotoxic cells (CTLs and NK cells) and tumor cells [22,23,24,25], and between DCs and tumor cells [26]. Interestingly, we previously reported that Cx43 expression is transcriptionally induced by HIF-1α in hypoxic melanoma cells [24]. However, whether hypoxic melanoma cells can transfer HRMs via Cx43-GJs to immune cells remains to be elucidated. In this report, using human and murine melanoma cell lines, we aimed to evaluate the involvement of Cx43 GJIC in the mobility of miRNAs induced by hypoxia, from melanoma cells to DCs and melanoma-specific CTLs. We identified that miR-192-5p was strongly induced in hypoxic melanoma cells, and it was acquired by CTLs and DCs after being co-cultured with hypoxic melanoma, via a cell contact and Cx43-dependent mechanism. Interestingly, the levels of miR-192-5p in CTLs co-cultured with hypoxic melanoma cells inversely correlated with their cytotoxic activity and ZEB2 mRNA expression, an immune-related and validated target of miR-192-5p. Altogether, our data suggest that hypoxic melanoma cells may suppress CTL cytotoxic activity by transferring hypoxia-induced mi-192-5p through Cx43-GJs. As far as we know, we reported, for first time, a Cx43-mediated miRNA transfer between hypoxic tumors and immune cells, and provided evidence that support a Cx43-dependent mechanism of immune-evasion used by hypoxic melanoma cells.

## 2. Results

### 2.1. DCs and CTLs Acquire RNA Molecules from Hypoxic Melanoma Cells through a Cx43-GJ-Dependent Mechanism

**Figure 1:**
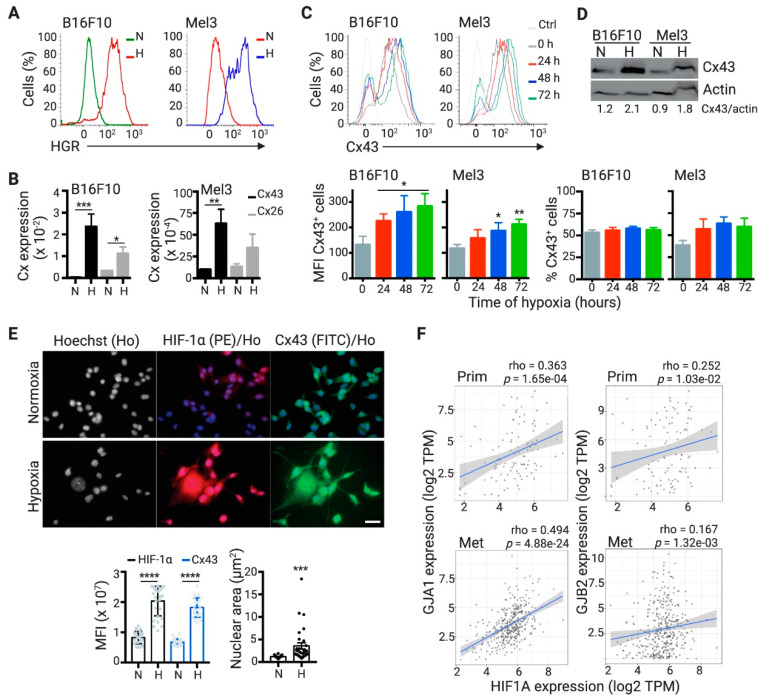
Hypoxia induces Connexin-43 (Cx43) expression in B16F10 and Mel3 melanoma cells. (**A**) Murine (B16F10) and human (Mel3) melanoma cells were cultured in normoxic (21% O_2_; N) or hypoxic (1% O_2_; H) conditions. For B16F10, 24 h of conditioned culture was used, while, for Mel3, 72 h of conditioned culture was used. Intracellular hypoxia was determined by hypoxia green reagent (HGR) staining. (**B**) Cx43 (gap junction protein alpha 1; Gja1 or GJA1) and Cx26 (gap junction protein beta 2; Gjb2 or GJB2) gene expression was determined in N and H B16F10 and Mel3 melanoma cells by qRT-PCR. The levels of Cx expression were obtained by the Δ/ΔCt method and normalized to actin expression. (**C**) Cx43 protein levels were determined by flow cytometry in melanoma cells cultured in the presence of 150 μM CoCl_2_ for different time points. (Upper) Representative histograms for Cx43 expression. Ctrl: antibody isotype control. Lower, bar graphs showing the Cx43 mean fluorescence intensity (MFI) (left) and frequency (right) in Cx43^+^ melanoma cells. (**D**) The expression of Cx43 and actin was assessed by Western blot in N and H melanoma cells cultured as described in (A). Cx43/actin ratios were quantified by ImageJ software. (**E**) B16F10 cells were cultured in absence (N) or presence (H) of 150 μM CoCl_2_ for 48 h. Hypoxia-inducible factor-1α (HIF-1α) (phycoerythrin; PE) and Cx43 (fluorescein isothiocyanate; FITC) protein levels were determined by immunofluorescence and confocal microscopy (representative images are shown). Nuclear staining was performed using Hoechst (Ho). Scale bar: 25 μm. Lower, quantification of HIF-1α and Cx43 MFI and nuclear area determination for approximately 30 cells per condition. * *p* < 0.05; ** *p* < 0.01; *** *p* < 0.001; **** *p* < 0.0001 (two-tailed Student’s *t*-test). (**F**) Correlation of GJA1 (left) or GJB2 (right) and HIF1A expressions in The Cancer Genome Atlas (TCGA) database estimated by TIMER2.0 resource (http://timer.cistrome.org/) in primary (Prim; *n* = 103 patients; upper) and metastasis (Met; *n* = 368 patients; lower) skin cutaneous melanoma. Partial spearman’s rho and *p* values are shown.

We observed that hypoxia induced Cx43 expression in human (Mel3) and murine (B16F10) melanoma cell lines, both at mRNA and protein levels (Figure 1A–D), supporting previous evidence in other human melanoma cell lines [24]. This phenomenon was evident in cells cultured in low O_2_ tension (1% O_2_) or in presence of CoCl_2_, a chemical simulator of hypoxia. In addition to Cx43, melanoma cells mostly express Cx26 [27], whose expression was only marginally responsive to hypoxia (Figure 1B). Hypoxia increases Cx43 expression in human melanoma cells via HIF-1α transcriptional activation [24]. Accordingly, CoCl_2_-stimulated B16F10 cells showed increased levels of both Cx43 and nuclear HIF-1α as compared with control normoxic cells (Figure 1E). Moreover, CoCl_2_-stimulated B16F10 cells showed augmented nuclear areas, a previously reported hypoxia-induced phenomenon in colon cancer cells [28]. Importantly, a strong positive correlation between the expression of HIF1A and GJA1 (encoding for HIF-1α and Cx43, respectively) was observed in a large cohort of metastatic melanoma patients (*n* = 368), described in The Cancer Genome Atlas (TCGA) database (Figure 1F). Associated with our in vitro results, this correlation was not observed for Cx26 (GJB2 gene) (Figure 1F).

**Figure 2:**
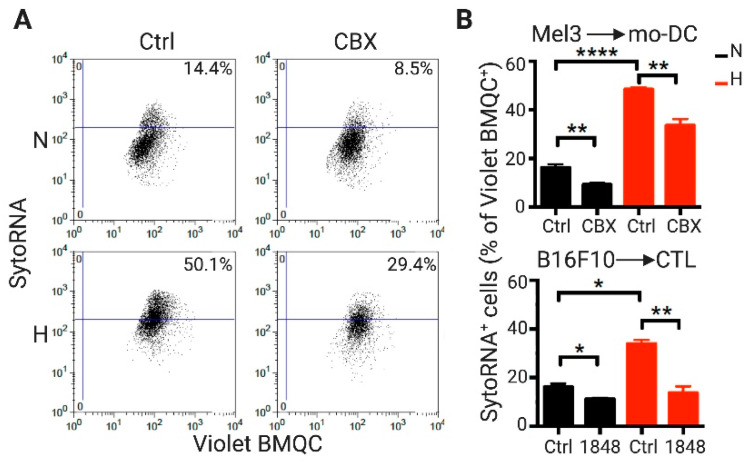
Human monocyte-derived dendritic cells and murine cytotoxic T lymphocytes acquire RNA molecules from hypoxic melanoma cells by a Cx43 gap junction-dependent mechanism. Mel3 human melanoma cells were cultured in normoxic (21% O_2_; N) or hypoxic (1% O_2_; H) conditions for 72 h, while B16F10 were cultured in the same conditions for 24 h. Intracellular RNA molecules were stained by cell incubation with SYTO RNASelect (green fluorescence dye; SytoRNA). Then, SytoRNA donor melanoma cells were co-cultured for 4 h with Violet BMQC [2,3,6,7-tetrahydro-9-bromomethyl-1H,5H-quinolizino(9,1-gh)coumarin)] pre-loaded human monocyte-derived dendritic cells (mo-DC) or pMEL-1 mouse cytotoxic T lymphocytes (CTL), respectively. Gap junction-mediated intercellular communications were inhibited using 50 μM of carbenoxolone (CBX) or Cx43 inhibitory mimic peptide 1848 (respective controls: Ctrl). The acquisition of green-fluorescent RNA by immune cells was determined by flow cytometry. (**A**) Representative dot plots for a Mel3/mo-DC co-culture. Violet BMQC^+^ cells were gated and the percentage of SytoRNA^+^ cells inside this population is shown. (**B**) The bar graphs show the percentage of Violet BMQC^+^ immune cells (mo-DC or CTL, upper and lower) that acquired RNA molecules from N or H Mel3 or B16F10 cells, respectively. * *p* < 0.05; ** *p* < 0.01; **** *p* < 0.0001 (one-way ANOVA, Tukey’s multiple comparison test).

As melanoma cells can form Cx43-mediated GJIC with CTLs [25], as well as with DCs [26], we tested whether hypoxia-mediated induction of Cx43 expression in melanoma cells correlated with higher levels of Cx43-GJ-mediated intercellular transfer of RNA molecules from melanoma to these immune cells. Hypoxic and normoxic Mel3 or B16F10 cells were treated with Syto RNASelect, which is a green fluorescent dye that labels RNA molecules [29], and were co-cultured with CellTracker Violet BMQC [2,3,6,7-tetrahydro-9-bromomethyl-1H,5H-quinolizino(9,1-gh)coumarin)] -pre-loaded human monocyte-derived DCs (mo-DCs) or gp100-specific CTLs differentiated from pMEL-1 mouse splenocytes, respectively. After 4 h of co-culture, the levels of fluorescent RNAs acquired by the Violet BMQC^+^ immune cells were determined by flow cytometry. Our results showed that hypoxic melanoma cells transferred more than twice the amount of RNA molecules to mo-DCs or CTLs than normoxic melanoma cells (Figure 2). Interestingly, and associated with increased Cx43 expression, transferred RNA levels were significantly reduced when the co-cultures were performed in presence of 50 μM carbenoxolone (CBX; chemical inhibitor of GJIC) or a Cx43 inhibitor mimetic peptide (1848) [30]. Therefore, our results suggest that hypoxia induces RNA transfer from melanoma cells to immune cells (DCs and CTLs) by a Cx43-GJ-dependent mechanism.

### 2.2. DCs Acquire Hypoxia-Induced miRNAs from Melanoma Cells by a Cx43-Dependent Mechanism 

**Figure 3:**
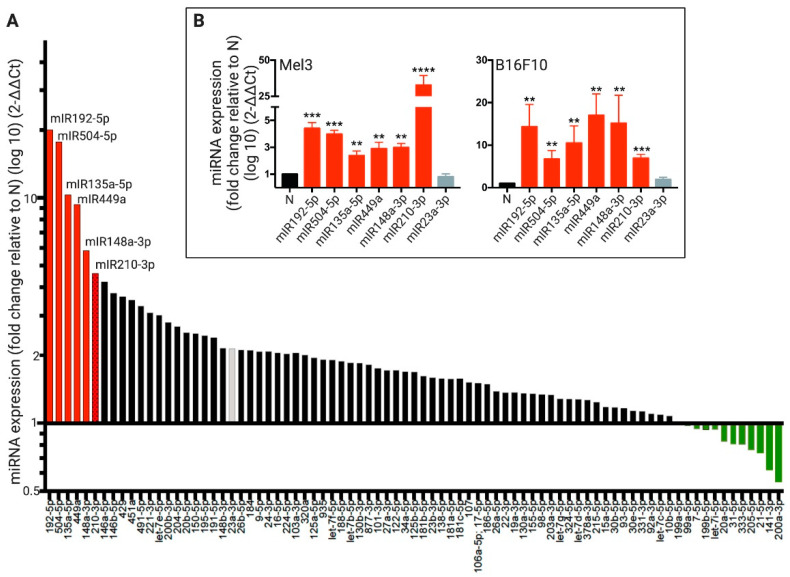
Identification of hypoxia-induced miRNAs in human and murine melanoma cells. (**A**) Human melanoma cells (Mel1, Mel2, and Mel3) were cultured in normoxic (21% O_2_; N) or hypoxic (1% O_2_; H) conditions for 72 h and total RNA samples (including miRNAs) were isolated. The expression levels of 84 hypoxia-associated miRNAs were determined by qRT-PCR arrays using pooled RNA samples. The levels of expression for each miRNA were obtained by the Δ/ΔCt method, normalized to the expression levels of different control small RNAs and expressed as fold change relative to N condition. The red bars show the top-6 hypoxia-induced miRNAs. The green bars show the hypoxia-down regulated miRNAs. (**B**) Mel3 cells were incubated in N or H for 72 h, while B16F10 were incubated in the same conditions for 24 h. Total RNA samples (including miRNAs) were isolated and the expression levels of miR-192-5p, -504-5p, -135a-5p, -449a, -148a-3p, and -210-3p, the top-6 hypoxia-induced miRNA, identified in (A), were evaluated by independent qRT-PCR assays. In addition, the expression level of miR-23a-3p, a miRNAs weakly induced by hypoxia identified by the qRT-PCR array (A, gray bar), also was evaluated by independent qRT-PCR assays. The levels of miRNA expression were obtained by the Δ/ΔCt method, normalized to small nuclear RNA RNU6-2 expression and expressed as fold change relative to N condition. ** *p* < 0.01; *** *p* < 0.001; **** *p* < 0.0001 (two-tailed Student’s *t*-test).

Taking into consideration that Cx43 channels are permeable to miRNAs, that Cx43-GJs have been implicated in the transfer of miRNAs in other cellular and pathophysiological contexts [31], and the finding that Cx43-GJ inhibition impaired the levels of RNA molecules transferred from hypoxic melanoma to immune cells (Figure 2), we hypothesized that hypoxic melanoma cells may transfer HRMs to immune cells via Cx43-GJs. In order to test this hypothesis, first, we identified HRMs in human melanoma cells by using a hypoxia signaling qRT-PCR array. Of the correctly amplified miRNA included in the qRT-PCR array, 29 (36%) had more than a 2-fold induction under hypoxia (Figure 3A). Using this method, we found that five miRNAs showed higher hypoxia-induced fold-changes than miR-210-3p, which is a miRNA signature of hypoxia [32]. Therefore, we choose the top-6 HRMs to further confirm its hypoxia induction in Mel3 and B16F10 cells, using independent qRT-PCR assays. These 6 HRMs (miR-192-5p, miR-504-5p, miR-135a-5p, miR-449a, miR-148a-3p, and miR-210-3p) showed increased levels of expression in both melanoma cell lines under hypoxic conditions (Figure 3B). By contrast, a moderately hypoxia-induced miRNA, miR-23a-3p (Figure 3A, grey bar), did not show significant hypoxia-responsiveness when it was tested in independent qRT-PCRs in Mel3 and B16F10 cells (Figure 3B, grey bars).

**Figure 4:**
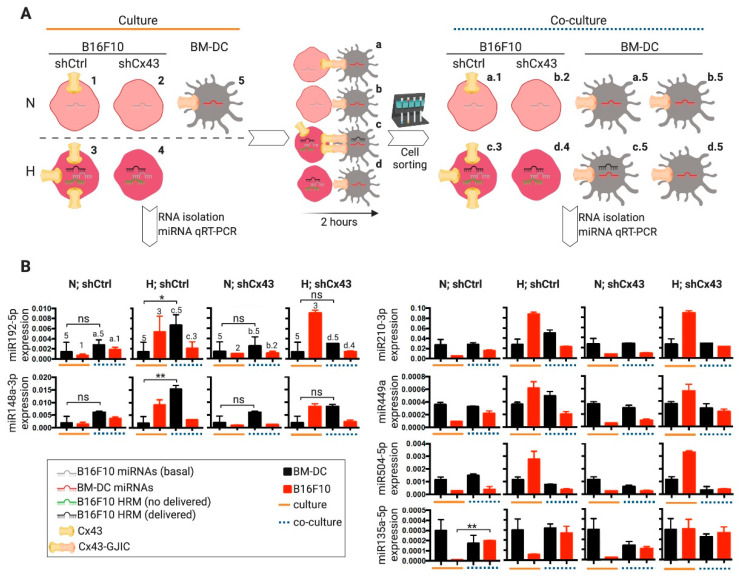
Bone marrow-derived dendritic cells acquire miR-192-5p and miR-148a-3p from hypoxic B16F10 melanoma cells by a Cx43-dependent mechanism. (**A**) Experimental scheme. B16F10 melanoma cells stably transfected with plasmids expressing short hairpin RNAs against Cx43 (shCx43) or control short hairpin (sh) RNAs (shCtrl), were cultured for 24 h upon normoxic (21% O_2_, N) or hypoxic (1% O_2_, H) conditions. Then, the N and H melanoma cells were co-cultured for 2 h with bone marrow-derived dendritic cells (BM-DC) upon a 1:2 cell ratio. After co-culture, BM-DCs were isolated from the co-culture by CD11c^+^ magnetic cell sorting, and CD11c^-^ melanoma cells were also collected. Total RNA samples (including the miRNAs) were isolated from pre-co-culture (named culture, orange lines) and post-co-culture (named co-culture, blue dashed lines) cells and miRNA levels were analyzed by qRT-PCR. (**B**) The levels of miR-192-5p, -148a-3p (**left**), -504-5p, -135a-5p, -449a, and -210-3p (**right**), were evaluated in the cells obtained as described in A (a number and letter code is showed for better comprehension). The miRNA levels were obtained by the Δ/ΔCt method and normalized to small nuclear RNA RNU6-2. Inset square: legend for (**A**) and (**B**). * *p* < 0.05; ** *p* < 0.01; ns: non-significant (two-tailed Student’s *t*-test).

To evaluate whether hypoxic melanoma cells transfer hypoxia-induced miRNAs to DCs, normoxic and hypoxic B16F10 cells were co-cultured with bone-marrow derived DCs (BM-DCs) for 2 h and then separated by CD11c magnetic cell sorting. The levels of the top-six HRMs were determined in each cell type before and after co-cultures (Figure 4A). After co-culture with hypoxic B16F10 cells, BM-DCs showed significantly higher levels of miR-192-5p and miR-148a-3p as compared with the pre-co-culture BM-DCs (Figure 4B, left). The levels of the remaining evaluated HRMs (miR-210-3p, miR-449a, miR-504-5p, and miR-135a-5p) showed a slight or no difference between BM-DC before and after co-culture with hypoxic B16F10 cells (Figure 4B, right). Importantly, the increasing on miR-192-5p and miR-148a-3p levels in post-co-cultured BM-DCs was not observed when Cx43 expression was silenced in B16F10 cells (Figure S1A) via stable transfection with plasmid expressing specific short-hairpin RNAs (shCx43) (Figure 4B), suggesting that hypoxic B16F10 cells transferred miR-192-5p and miR-148a-3p via a Cx43-dependent mechanism.

Our results also suggest that the transfer of miRNAs between hypoxic B16F10 and BM-DCs not only depends on their differential levels between the interacting cells, given that although miR-504-3p levels were higher in hypoxic B16F10 cells, there were no increase in its levels in BM-DCs after co-culture (Figure 4B). In addition, our results suggest that the transfer of miRNAs among BM-DCs and melanoma cells via Cx43 channels could be bi-directional, as it seems that normoxic B16F10 acquired miR-135a-5p from BM-DCs (Figure 4B). We also observed a similar phenomenon of miRNA transfer between human Mel3 melanoma cells and human mo-DCs. In this case, our results indicated that mo-DCs acquired miR-210-3p and miR-449a from hypoxic Mel3 cells (Figure S2).

### 2.3. CTLs Acquire miR-192-5p from Hypoxic Melanoma Cells by a Cell Contact and Cx43-Dependent Mechanism

**Figure 5:**
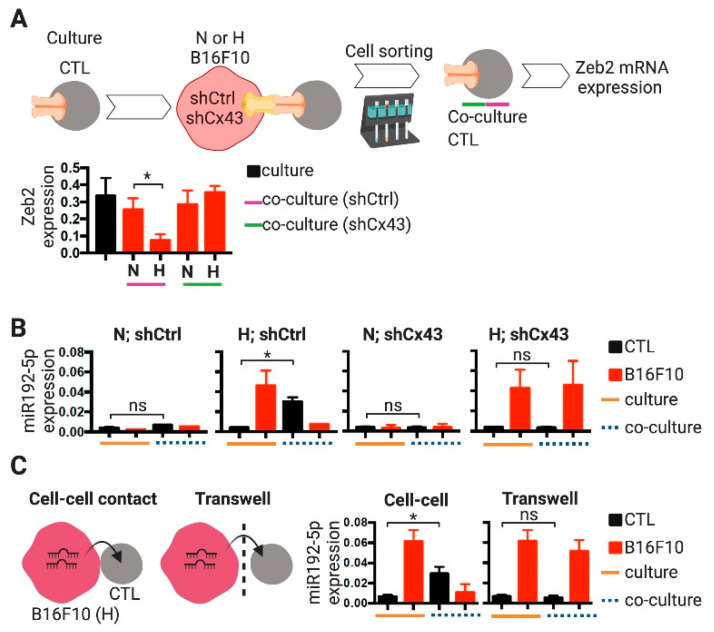
Cytotoxic T lymphocytes acquire miR192-5p from hypoxic B16F10 melanoma cells by a cell contact and Cx43-dependent mechanism. (**A**) Hypoxic B16F10 melanoma cells repress ZEB2 expression in cytotoxic T lymphocytes (CTLs). B16F10 melanoma cells stably transfected with plasmids expressing short hairpin RNAs against Cx43 (shCx43, green line) or control shRNAs (shCtrl, purple line), were cultured for 24 h upon normoxic (21% O_2_, N) or hypoxic (1% O_2_, H) conditions. Then, the N or H melanoma cells were co-cultured for 2 h with pMEL-1 CTLs upon a 1:2 cell ratio. After co-culture, CTLs were isolated by CD8^+^ magnetic cell sorting. Total RNA samples were isolated from pre-co-culture (named culture, black bar) and post-co-culture (named co-culture, red bars) CTLs and ZEB2 expression was analyzed by qRT-PCR. The levels of ZEB2 mRNAs were obtained by the Δ/ΔCt method and normalized to actin mRNA. (**B**) B16F10 cells stably transfected with plasmids expressing shCx43 or shCtrl, were cultured for 24 h upon N (21% O_2_) or H (1% O_2_) conditions. Then, the N or H melanoma cells were co-cultured for 2 h with pMEL-1 CTL upon a 1:2 cell ratio. After co-culture, CTLs were isolated by CD8^+^ magnetic cell sorting, and CD8^-^ melanoma cells were also collected. Total RNA samples (including the miRNAs) were isolated from pre-co-culture (named culture, orange lines) and post-co-culture (named co-culture, blue dashed lines) cells and miR-192-5p levels were analyzed by qRT-PCR. miR-192-5p expressions were obtained by the Δ/ΔCt method and normalized to small nuclear RNA RNU6-2. (**C**) Hypoxic B16F10 cells were co-cultured for 2 h with pMEL-1 CTL upon a 1:2 cell ratio allowing or not (transwell) cell-to-cell contact. After co-culture, CTLs were isolated by CD8^+^ magnetic cell sorting (CD8^-^ melanoma cells were also collected) and miR-192-5p expression was analyzed by qRT-PCR as described in (**B**). * *p* < 0.05; ns: non-significant (two-tailed Student’s *t*-test).

A search in the miRTarBase 2020 database [33] showed that all the strongly validated mouse targets for miR-192-5p and miR-148a-3p have relevant immune functions in DCs or T cells (Table 1). The only strongly validated target for murine miR-192-5p that showed up in this search was ZEB2 [34]. This transcription factor, best known for driving epithelial to mesenchymal transition through the repression of epithelial genes, has also important roles in regulating transcriptional networks necessary for DC and T cell differentiation, maintenance and function [35]. Indeed, ZEB2 regulates the expression of many important genes (like GZMA, coding for granzyme a) necessary for the formation of the terminal effector and effector-memory states in CD8^+^ T cells [36,37]. Accordingly, we observed that, after co-culture with hypoxic but not with normoxic B16F10 cells, pMEL-1 CTLs expressed significantly lower levels of ZEB2 (Figure 5A). Interestingly, this hypoxic B16F10-mediated repression of CTL ZEB2 expression was not observed when melanoma Cx43 expression was down-regulated by Cx43-shRNAs (Figure 5A). These results suggest that, as we showed for BM-DCs (Figure 4B), hypoxic B16F10 cells may transfer miR-192-5p (that targets ZEB2) to CTLs by a Cx43-mediated mechanism.

To determine whether hypoxic B16F10 cells can transfer miR-192-5p to gp100-specific CTLs by a Cx43-mediated mechanism, normoxic and hypoxic shCtrl- or shCx43-expressing B16F10 cells were co-cultured with pMEL-1 CTLs for 2 h, separated by CD8 magnetic cell sorting and miR-192-5p levels were determined in each cell type. After co-culture with hypoxic B16F10 cells, CTLs showed significantly increased miR-192-5p levels, compared with pre-co-culture CTLs (Figure 5B). Importantly, these augmented levels of miR-192-5p in post-co-cultured CTLs were not observed when the hypoxic B16F10 expressed shCx43, suggesting that hypoxic B16F10 cells transferred miR-192-5p to CTLs via a Cx43-dependent mechanism (Figure 5B). The transfer of miRNAs among cells could be mediated by EV (mainly by exosomes) [18] that also contain functional Cx43 channels [44], and/or by cell-cell GJIC [18]. Our results also suggest that hypoxic B16F10 cells transfer miR-192-5p to CTLs via a cell-to-cell contact-dependent manner, given that the levels of this miRNA did not changed in CTLs after transwell co-cultures with B16F10 cells (Figure 5C).

**Figure 6:**
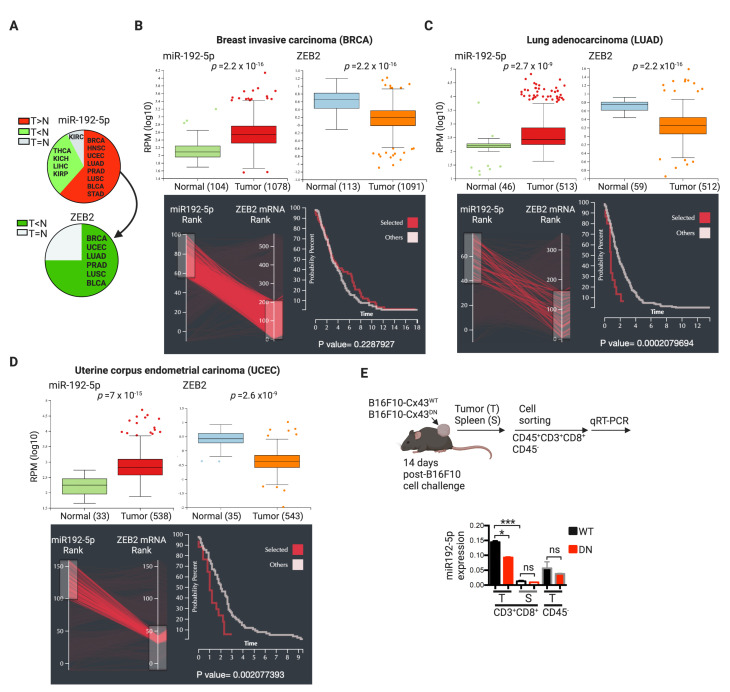
miR-192-5p levels are abundant in human tumors and are higher in CTLs infiltrating B16F10 tumors expressing functional Cx43 channels. (**A**) miR-192-5p is significantly more abundant in 8 out of 13 tumors (T), as compared with the corresponding normal (N) tissue in large cohorts of cancer patients described in The Cancer Genome Atlas (TCGA) dataset analyzed by miRNA Target Viewer (miR-TV) resource (http://mirtv.ibms.sinica.edu.tw). Six of these eight tumors have an inverse pattern for ZEB2 mRNA expression. (**B**–**D**) Expression levels of miR-192-5p and its target ZEB2 (mRNA) in tumors and adjacent normal tissues in three different cancer types described in the TCGA database analyzed by miR-TV resource. Below, each box plot shows the Kaplan-Meier survival curves for patients with the highest miR-192-5p and lower ZEB2 expression (red lines; “selected”), versus the rest (light gray lines, “others”). HNSC: head and neck squamous cell carcinoma; PRAD: prostate adenocarcinoma; LUSC: lung squamous cell carcinoma; BLCA: bladder urothelial carcinoma; STAD: stomach adenocarcinoma; KIRC: kidney renal clear cell carcinoma; THCA: thyroid carcinoma; KICH: kidney chromophobe; LIHC: liver hepatocellular carcinoma; KIRP: kidney renal papillary cell carcinoma. (**E**) C57BL6 mice were challenged subcutaneously with B16F10 cells expressing wild type or a dominant negative Cx43 mutant (Cx43^WT^, Cx43^DN^, respectively). Tumors (T) and spleens (S) were collected 14 days after tumor challenging, and CD8^+^ T cells and non-immune (CD45^−^) cells were isolated by fluorescence-activated cell sorting (FACS). RNA samples were obtained from the isolated cells and miR-192-5p expression was analyzed by qRT-PCR. * *p* < 0.05; *** *p* < 0.001; ns: non-significant (one-way ANOVA, Tukey’s multiple comparison test).

Interestingly, using the interactive web resource for miRNA-target gene expression analysis miR-TV (miRNA Target Viewer) [45], we found that miR-192-5p is significantly more abundant in 8 out of 13 tumors, as compared with the corresponding normal tissues in large cohorts of cancer patients described in the TCGA dataset (Figure 6A). In six of these eight cancers, an inverse pattern for ZEB2 mRNA expression was also observed (i.e., lower expression in tumors as compared with normal tissues) (Figure 6A–D). Out of these six, we chose the three cancer types with the highest statistical significance in tumor/normal tissue for miR-192-5p and ZEB2 differential expression (breast invasive carcinoma, BRCA; uterine corpus endometrial carcinoma, UCEC; and lung adenocarcinoma, LUAD) and analyzed the survival time of patients according to the miR-192-5p/ZEB2 expression patterns. Interestingly, we observed that higher and lower expressions of miR-192-5p and ZEB2, respectively, were associated with poorer survival rates in patients with UCEC (*p* = 0.002) and LUAD (*p* = 0.0002) (Figure 6B–D). Moreover, and in agreement with the role of ZEB2 in the expression of terminal effector genes in CD8^+^ T cells [35], we observed stronger positive correlations between the expression of ZEB2 and GZMA and GZMB (encoding for granzyme A and B, respectively) in BRCA, UCEC, and LUAD patients (Figure S3).

**Figure 7:**
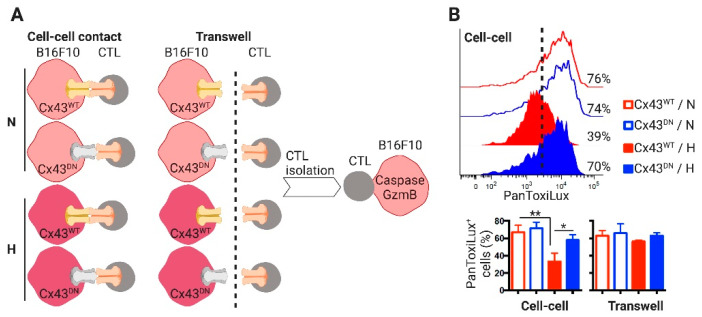
Hypoxic B16F10 cells suppress cytotoxic activity of cytotoxic T lymphocytes by a Cx43- and cell contact-dependent mechanism. (**A**) Experimental scheme. B16F10 melanoma cells expressing wild type or a dominant negative Cx43 mutant (Cx43^WT^, Cx43^DN^, respectively) were cultured for 24 h upon normoxic (21% O_2_, N) or hypoxic (1% O_2_, H) conditions. Then, N or H melanoma cells were co-cultured for 2 h with pMEL-1 cytotoxic T lymphocytes (CTL) upon a 1:2 cell ratio allowing or not (transwell) cell-to-cell contact. After co-culture, CTLs were collected and co-cultured with PanToxiLux (fluorogenic caspase and granzyme B substrate)-pre-loaded B16F10 cells. The cytotoxic activity of the CTLs was determined by flow cytometry. (**B**) (Upper) representative histograms showing PanToxiLux fluorescence (as % of PanToxiLux^+^ cells) in B16F10 target cells. Lower, the bars graph shows the percentage of PanToxiLux^+^ cells in the different co-cultures. * *p* < 0.05; ** *p* < 0.01 (one-way ANOVA, Tukey’s multiple comparison test).

In order to determine if the Cx43-mediated transfer of miR-192-5p from B16F10 cells to CD8^+^ T cells could occur in the TME, we challenged C57BL6 mice with B16F10 cells expressing wild type (Cx43^WT^) or dominant negative (Cx43^DN^) mutant Cx43 that causes closed channels [46]. As expected, B16F10 cells expressing the Cx43^DN^ established lower levels of GJIC with CTLs as compared with control cells (Figure S1B). Fourteen days after the B16F10 cell challenge, we evaluated the expression of miR-192-5p in tumor infiltrating and splenic CD8^+^ T cells and in non-immune (CD45^−^) cells. Tumor infiltrating CD8^+^ T cells showed higher levels of miR-192-5p than CD8^+^ T cells from the spleens and, interestingly, these levels were significantly lower in CD8^+^ T cells isolated from Cx43^DN^-expressing than from Cx43^WT^-expressing melanoma tumors (Figure 6E). These results suggest that, as observed in co-cultured cells, CTLs may acquire miR-192-5p from B16F10 cells by a Cx43-dependent mechanism in vivo. Interestingly, and associated with the levels of miR-192-5p and ZEB2, the hypoxic B16F10 cells suppressed the cytotoxic activity of pMEL-1 CTLs by a Cx43- and cell contact-dependent mechanism (Figure 7). Our results indicate that after cell-contact-permissive co-cultures with hypoxic B16F10-Cx43^WT^ cells, pMEL-1 CTLs triggered less caspase and granzyme b activities in target melanoma cells than after co-culture with normoxic or hypoxic B16F10-Cx43^DN^ cells (Figure 7). All these data suggest that the miR-192-5p/ZEB2 axis may have an important role in the regulation of CTL-mediated tumor immunity, particularly in the context of hypoxic TME.

## 3. Discussion

Recent advances in immunotherapeutic approaches have raised unprecedented clinical achievements in the treatment of cancer, particularly in melanoma [47]. However, a considerable fraction of treated patients remain as non-responders and/or acquire resistance to immunotherapy [48]. Moreover, the induction of tumor-specific immune responses by immunotherapies is not always followed by tumor rejection (objective response) and long-term patient survival, mainly because cancer cells acquire the capacity to avoid normalizing cues from their microenvironment, including evasive mechanisms that render them resistant to immune cytotoxic attack. In this line, since more than five decades ago, it is known that cancer cells isolate themselves from their cellular environment down-regulating GJIC [49]. Indeed, aberrant Cx43 expression and its mislocalization, which leads to reduced GJIC, are frequent in different tumors, including melanoma, and predict cancer patient outcome [19,50]. Numerous evidences have shown that Cx43 suppresses tumors via different heterotypic (cancer cell-immune cell or cancer cell-stromal cell) GJICs, as we previously reviewed [19,23]. Indeed, Cx43-CJICs have been implicated in different antitumor roles that affect the cancer-immunity cycle (Figure S4). For example, it has been observed that Cx43-GJICs increase tumor immunogenicity via STING-mediated production of type I interferon (IFN) by colorectal tumor associated-DCs that acquire cancer cell-generated cyclic guanosine monophosphate-adenosine monophosphate (cGAMP) by Cx43-GJs [51].

Nevertheless, recent evidence also indicates that GJICs and Cxs could also promote cancer progression and tumor aggressiveness, depending on cancer type, disease stage, and Cx isotype [52]. Indeed, depending on these variables, expression of Cxs in tumor biopsies could be associated with good or bad prognosis in cancer patients [19]. Therefore, Cxs are currently considered as conditional tumor suppressor genes. In fact, heterotypic Cx43-GJICs among cancer cells and immune cells can also promote tumor progression [21]. For example, Cx43-GJs allow brain metastatic cancer cells transferring cGAMP to astrocytes, leading to the activation of the STING pathway and the subsequent production of IFN-α and tumor necrosis factor by the cGAMP-receiving astrocytes [53]. These cytokines then lead to paracrine activation of STAT1 and NF-κB pathways in brain metastatic cells, supporting tumor growth and chemoresistance [53]. This pro-tumor role of Cx43-GJ-mediated cGAMP transfer contrasts with its anti-tumor activities observed in colorectal cancer [51], highlighting the aforementioned context-dependent role of Cx43 in cancer immunity and tumor progression.

Interestingly, hypoxia, a microenvironmental condition that promotes tumor progression and immune evasion, also modulates Cx43 expression and function in cancer and other biological contexts. Interestingly, recent data shows a significantly decrease in Cx43 expression in the left ventricular of HIF-1α conditional knockout mice, which may contribute to impaired heart contractility [54]. This HIF-1α-dependent Cx43 expression in cardiac cells supports our previous evidence showing that HIF-1α binds GJA1 promoter, inducing Cx43 expression in hypoxic melanoma cells [24]. In the present study, we confirmed that hypoxia promotes Cx43 expression in additional human and mouse melanoma cell lines. Interestingly, our data also suggest that this phenomenon could occur in patients, given the positive correlation of HIF1A and GJA1 expressions observed in metastatic melanoma TCGA dataset (Figure 1). Additional reports also indicate that hypoxia modulates Cx43 and GJIC in other cancer types. For example, it has been shown that hypoxia induces Cx43 expression and GJIC in pancreatic ductal adenocarcinoma cells, promoting tumor progression by different metabolic cell coupling-mediated mechanisms [55,56]. We have previously identified that the pre-incubation of melanoma cells to hypoxic conditions, decreases their susceptibility to NK cell cytotoxic attack by interfering with Cx43-GJICs via autophagy-mediated Cx43 degradation at the cytotoxic immunological synapse [24]. In the present report, we observed that hypoxic melanoma cells can establish more Cx43-mediated GJICs with DCs and CTLs than normoxic melanoma cells (Figure 2), allowing the transfer of specific hypoxia-induced miRNAs (HRMs) from tumor to immune cells (Figure 4 and Figure 5). Moreover, we generated evidence that suggests that this kind of tumor cell Cx43-dependent modulation of miRNA content in CTLs could also occur in vivo (Figure 6).

The first studies suggesting a role of Cx43-GJs in the intercellular transfer of functional RNA molecules were performed using exogenously introduced small interfering (siRNA) or shRNAs [57,58]. Similarly, Katakowski and collaborators showed that the delivery of functional and exogenously introduced miRNAs between rat gliosarcoma cells could be inhibited by CBX [59]. Interestingly, Cx43 channels have higher permeability to different miRNAs than channels constituted by other Cxs, like Cx26, Cx30, and Cx31 [60]. Given the cell-specific expression of Cxs, this differential permeability of different Cxs may have important implications in the non-cell autonomous miRNA-mediated gene regulation in different cells. Additional reports have also suggested a physiological role for the GJ-mediated miRNA shuttling, including in the context of cancer. For example, bone marrow stromal cells can transfer miR-127, miR-197, miR-222, and miR-223 to breast cancer cells via GJ channels, targeting CXCL12 expression, inhibiting cancer cell proliferation and impacting metastatic dormancy [61]. Likewise, human macrophages can deliver miR-142 and miR-223 to hepatocarcinoma cells by a contact-dependent mechanism that is inhibited by different GJ chemical blockers [62]. These miRNAs targeted the expression of stathmin-1 and insulin-like growth factor-1 receptor in the tumor cells, functionally inhibiting their proliferation level [62]. More recently, it was reported that the inhibition of glioma cell proliferation by miR34a can be increased by allowing its transfer via Cx43-GJs, which suggests that antitumor miRNAs can be combined with Cx43 enhancers in order to improve miRNA therapeutic effects [31].

The deregulation of miRNA expression has been implicated in tumor initiation, progression and resistance to therapy or immune attack in most cancers, including in melanoma [63]. Many of these miRNA deregulations are a result of adaptation pathways to hypoxia and/or are regulated by HIF-1α [64]. Some of these hypoxia-induced miRNAs have been implicated in cancer progression, promoting immune-resistance phenotypes in cancer cells. For example, in a very elegant study, Wu and collaborators showed that two hypoxia-induced miRNAs, miR-25 and miR-93, repressed cyclic GMP-AMP synthase (cGAS) expression during hypoxia, via targeting the epigenetic factor NCOA3 in breast cancer cells, which lead to hypoxic tumor cells to escape immunological responses induced by damage-associated molecular patterns [65]. As mentioned before, HRMs can also modulate immune cells of the TME via non-cell-autonomous mechanisms, including the direct mobilization of the miRNAs in EV. Very interestingly, it has been reported that EV, mainly exosomes, contain functional Cx43-channels that allow the exchange of material between exosomes and recipient cells [44,66]. In addition, a putative role for Cx43 channels in the recruitment of miRNA into exosomes has also been proposed, given that Cx43 contains several RNA-binding motifs [67]. Another Cx, Cx46, which is strongly induced by hypoxia in breast cancer cells [68], was recently observed to be contained as functional channels in breast cancer derived EV [69]. These antecedents suggest that Cx43 channels may also participate in the transfer of hypoxia-induced miRNAs, contained in tumor derived EV, to immune cells. Although our results indicate a cell-contact dependence in the Cx43-mediated transfer of miR-192-5p from melanoma to CTLs, we cannot discard the participation of melanoma Cx43^+^ exosomes in the transfer of this (and/or others) miRNA in different experimental conditions. 

Our results also suggest that the identity of the transferred miRNAs depends on the nature of the interacting cells more than the levels of miRNAs in those cells. In our experimental conditions, miR-192-5p was transferred from hypoxic melanoma cells to DCs, as well as to CTLs. The hypothetical functional relevance of these observations was suggested by the analysis of the expression of a strongly validated miR-192-5p target, ZEB2, a transcription factor that regulates the expression of many important genes (like GZMA) necessary for the formation of the terminal effector a state in CD8^+^ T cells [36,37]. Accordingly, we observed that after co-culture with hypoxic B16F10 cells, CTL have elevated levels of miR-192-5p, decreased levels of ZEB2 and low cytotoxic activity. Interestingly, we also observed that cancer patients with this expression pattern have poor prognosis, indicating that the miR-192-5p/ZEB2 axis could be relevant in the immune evasion of tumors during cancer progression. Although we do not provide mechanistic data of the effects of miR-192-5p in the recipient immune cells, recent evidence has shown that miR-192 contained in circulating EV regulates cytokine expression in EV-receiving macrophages [70]. In addition, other report has shown that miR-192 suppresses CD4^+^ T follicular helper cell differentiation by targeting CXCR5 [71]. Whether miR-192-5p directly targets ZEB2 (or other mRNA genes) on DCs and CTLs remains to be elucidated in further investigations. Finally, as far as we know, we reported, for the first time, a Cx43-mediated miRNA transfer between hypoxic tumors and immune cells, and provided evidence that support a Cx43-dependent mechanism of immune-evasion used by hypoxic melanoma cells (Figure S5). Altogether, this evidence can be useful for the designing of target therapies aimed to improve immunotherapy outcomes for hypoxic cancers. 

## 4. Materials and Methods

### 4.1. Mice

Wild-type C57BL6 and transgenic pMEL-1 (C57BL6 background) mice [72] were bred at Bioterio de alta seguridad (BAS) at Universidad de Chile. For all experiments, mice between 6 and 12 weeks of age were bred in specific pathogen-free conditions. All animal experiments were performed in accordance with institutional guidelines for animal care and were approved by the Ethical Review Committees at the Faculty of Medicine of the Universidad de Chile, ethical number: CBA FMUCH 0906 (approval date: 13 December 2016).

### 4.2. Cell Lines

B16F10 (ATCC^®^ CRL-6475™) and Mel1, Mel2 and Mel3 [73] melanoma cell lines were maintained in RPMI-1640 medium supplemented with 10% fetal bovine serum (FBS) and 1% streptomycin/penicillin (all from Corning, Manassas, VA, USA) at 37 °C under 5% CO_2_ and 95% relative humidity.

### 4.3. Hypoxia Treatments 

For the induction of hypoxia, melanoma cell cultures were incubated for 24 (B16F10) to 72 h (Mel3) in a Modulator Incubator Chamber (Billups-Rothenberg, Inc., San Diego, CA, USA) in a humidified atmosphere containing 5% CO_2_, 1% O_2_, and 94% N_2_ (special gas cylinder supplied by Linde Chile) at 37 °C. In some experiments, CoCl_2_ (Sigma-Aldrich, St. Louis, MO, USA), a chemical inductor of hypoxia, was used at 150 μM final concentration. Hypoxia induction was routinely tested by flow cytometry using Hypoxia Green Reagent (HGR, Invitrogen, Paisley, UK) according to the manufacturer instructions. HGR is a membrane-permeant probe that releases rhodamine as O_2_ levels decrease, resulting in a fluorogenic response.

### 4.4. pMEL-1 CTL Differentiation 

pMEL-1 CTLs were differentiated as described before [25]. Briefly, total splenocytes of pMEL-1 mice were obtained by perfusion of spleens and then cultured for six days in RPMI GlutaMAX™ culture medium supplemented with 10% FBS (HyClone™, South Logan, UT, USA), 1% streptomycin/penicillin (Corning), and 55 μM-mercaptoethanol (Gibco, Carlsbad, CA, USA) and stimulated with gp100 peptide (1 μM KVPRNQDWL; Genetel Laboratories LLC, Madison, WI, USA) and recombinant human (rh) IL-2 (30 IU/mL; Miltenyi Biotec, Bisley, UK). Fresh culture medium with rhIL-2 was added on days 2 and 4. At day 6, the phenotype of the cells was analyzed by flow cytometry as described before [25].

### 4.5. moDC and BM-DC Differentiation

Murine BM-DCs were differentiated from femurs and tibias of C57BL/6 mice by culturing bone marrow cells in the presence of 20 ng/mL mouse recombinant GM-CSF (Biolegend) for 8 days. Fresh culture medium with cytokine was added on day 3, and, on day 6, the medium was refreshed. The phenotype of the cells was analyzed by flow cytometry as described before [74]. Human peripheral blood moDCs were differentiated from peripheral blood mononuclear cells (PBMCs), as described before [73].

### 4.6. Total RNA Transfer Assay

Mel3 or B16F10 melanoma cells were cultured in normoxic (21% O_2_) or hypoxic (1% O_2_) conditions for 72 (Mel3) or 24 h (B16F10). Intracellular total RNA molecules were stained by incubating the melanoma cells with 5 μM Syto RNASelect (Invitrogen, Paisley, UK) [29] for 20 min and washed according to manufacturer’s instructions. mo-DC and pMEL-1 mouse-derived CTLs were loaded with the CellTracker Violet BMQC for 10 min according to manufacturer’s instructions (10 μM for DCs and 2.5 μM for CTLs; Invitrogen), the reaction was stopped by adding FBS for 1 min, and then the cells were washed. SytoRNA donor Mel3 or B16F10 cells were co-cultured for 4 h with the Violet BMQC preloaded mo-DC or pMEL-1 CTLs, respectively, at a 1:3 ratio. GJs were inhibited including in the co-cultures 50 μM of CBX (Sigma-Aldrich, St. Louis, MO, USA), a Cx43 inhibitory mimic peptide named 1848 (300 μM), or its respective controls (Ctrl), vehicle or gap20 peptide, as described before [21]. After co-culture, the acquisition of RNA in Violet BMQC^+^ recipient cells was determined by flow cytometry.

### 4.7. miRNA Transfer Assays

Melanoma cells were cultured in normoxic and hypoxic conditions for 24 (B16F10, shCx43-B16F10, shCtrl-B16F10) or 72 h (Mel3). Then, 1 × 10^5^ melanoma cells were co-culture with BM-DC, pMEL-1 CTLs or moDC at 1:2 cell ratios for 2 h. After co-culture, CD11c^+^ cells (BM-DC, moDC) or CD8^+^ cells (CTL) were isolated from CD11c^-^ or CD8^-^ cells (melanoma) using specific magnetic cell sorting according to manufacturer’s instructions (MACS, Miltenyi Biotec, Bisley, UK). Total RNA samples, including miRNA, were isolated from pre-co-culture (named culture) and post-co-culture (named co-culture) using miRNeasy Mini kit according to manufacturer’s instructions (Qiagen, Hilden, Germany). Quality and concentration of RNA samples were determined by Agilent 2200 TapeStation analysis using RNA ScreenTapes according to manufacturer instructions (Agilent Technologies, Santa Clara, CA, USA). Only RNA samples with RNA integrity number (RIN) values >8 were used. cDNAs were generated by reverse transcription using miScript II RT Kit and the miScript HiFlex buffer according to manufacturer instructions (Qiagen). Expressions of different mature miRNAs were determined using miScript SYBR Green PCR Kit and specific mouse or human miScript primer assays according to the manufacturer’s protocols (Qiagen). Levels of each miRNA were expressed relative to the level of small nuclear RNA RNU6-2 (miScript primer assays) and analyzed according to the Δ/ΔCt method. PCRs were performed in a LightCycler 480 Instrument and LightCycler 480 Software (Roche, Basel, Switzerland). As controls of mature miRNA transfer or cell isolation, precursor miRNA expression levels or CD8 and gp100 mRNA expressions were determined in culture and co-cultured cells, using specific miScript primer assays (for precursor miRNAs) or designed primers for mRNA (see Section 4.12) (Figure S6).

For in vivo determination of miR-192-5p expression, 6–12 weeks old C57BL6 mice were subcutaneously challenged with 1.5 × 10^5^ B16F10 cells expressing wild type (Cx43^WT^) or a dominant negative Cx43 mutant (Cx43^DN^). Fourteen days after tumor challenge, spleens and tumors were collected and treated as described before [74] and non-immune (CD45^−^) and CD45^+^ CD3^+^ CD8^+^ T cells were isolated using FACS sorting. RNA samples were obtained and miR-192-5p expression analyzed as described before.

### 4.8. Pantoxilux Cytotoxic Assay

B16F10 cells expressing wild type (Cx43^WT^) or a dominant negative Cx43 mutant (Cx43^DN^) were cultured for 24 h upon normoxic or hypoxic conditions. Then, pre-conditioned melanoma cells were co-cultured for 2 h with pMEL-1 CTLs at a 1:2 cell ratio, allowing or not (Transwell plates, Corning) cell-to-cell contact. After co-culture, the cytotoxic activity of the CTLs against B16F10 cells was determined by flow cytometry using the Pantoxilux^®^ (OncoImmunin, Inc., Gaithersburg, MD, USA) granzyme and upstream caspase activity kit, following manufacturer’s instructions.

### 4.9. Flow Cytometry

Flow cytometry experiments were performed as previously described [30,74]. Intracellular hypoxia was determined by hypoxia green reagent (HGR) staining, according to manufacturer’s instructions (Thermofisher, Waltham, MA, USA). To detect Cx43, a rabbit polyclonal anti-human/mouse Cx43 antibody, directed to the C-terminal domain (C6219; Sigma-Aldrich), and a secondary donkey anti-rabbit fluorescein isothiocyanate (FITC)-conjugated antibody (Poly4064; BioLegend, San Diego, CA, USA) were used. The following monoclonal antibodies were used for CTL staining: CD8-V500 (dilution 1:500; clone 53-6.7), CD25-APC (dilution 1:1500; clone PC61), CD44- phycoerythrin (PE)-Cy7 (dilution 1:500; clone IM7), Vβ13-V450 (dilution 1:400; clone MR 12.3), all from BioLegend. 7-AAD (1:1500; BioLegend) staining was used for dead cell exclusion. For mo-DC phenotype evaluation, the following antibodies were used: IA/IE-APC-Cy7 (dilution 1:1500; clone M5/114.15.2; Biolegend), CD11c-APC (dilution 1:800; clone N418; eBioscience™). LIVE/DEAD™ Fixable Aqua (dilution 1:800; Thermofisher) was used for dead cell exclusion. Samples were acquired on a FACSVerse. (BD Biosciences, Hershey, PA, USA) and analyzed using the FlowJo software (version 10.0.7; Tree Star Inc., Ashland, OR, USA). For CD8^+^ and CD45^−^ FACS sorting, the following monoclonal antibodies were used: CD3-AlexaFluor488 (dilution 1:400; clone 17A2), CD8-PE (dilution 1:200; clone 53-6.7), CD45-PerCP-Cy5.5 (dilution 1:400; clone 30-F11), all from BioLegend. 7-AAD (1:1500; BioLegend) staining was used for dead cell exclusion.

### 4.10. Microscopy

Confocal microscopy of fixed B16F10 cells was performed as previously described [24]. Briefly, B16F10 cells were cultured overnight at 37 °C and 5% CO_2_ on poly-l-lysine-coated slides, using 5 × 10^4^ cells per slide in 100 μL of complete culture medium. Cells were then cultured in absence (N) or presence (H) of 150 μM CoCl_2_ (Sigma-Aldrich) for additional 48 h and gently washed twice with phosphate-buffered saline (PBS) and fixed with 4% paraformaldehyde for 15 min at room temperature in the dark. After gentle washing with PBS, cells were incubated in ammonium chloride (50 mM) for 10 min. Then, cells were permeabilized for 10 min (0.5% Triton X-100 and 0.5% FBS) and blocked with 0.5% bovine serum albumin (BSA) for 15 min. Cells were stained with 1:500 dilutions of FITC-coupled monoclonal antibody anti-Cx43 (D-7; Santa Cruz Biotechnology, Dallas, TX, USA) or a PE-coupled monoclonal antibody anti-HIF-1α (28b, Santa Cruz Biotechnology) for 1.25 h at room temperature, and then stained with 1:1000 dilution of Hoechst 33,342 (Invitrogen) for 15 min. Cells were analyzed with a C2+ confocal microscope (1000, Nikon Instruments Inc., Melville, NY, USA) and a Spinning Disk Olympus BX61WI microscope (400, Center Valley, PA, USA). Cx43 and HIF-1α expression and nucleus areas were quantified using ImageJ software (version number 1.48v, National Institutes of Health, Bethesda, MD, USA).

### 4.11. Transfections

For knocking down Cx43 expression, B16F10 and Mel3 melanoma cells were stably transfected with lentiviral vector plasmids encoding a pool of 4 Gja1 (sc-35091-SH) or GJA1 (sc-29276-SH)-specific shRNAs (plus hairpin), using plasmid transfection reagent (sc-108061) and shRNA plasmid transfection medium (sc-108062) according to the manufacturer’s instructions (Santa Cruz Biotechnology). A control shRNA plasmid (sc-108060, Santa Cruz Biotechnology) was used for generating stable transfection in the control cells. Puromycin (5 μg/mL; sc-108071, Santa Cruz Biotechnology) was added to the culture medium for selection of transfected cells.

B16F10 cells were stably transfected with pcDNA3.1 + NeGFP plasmids containing wild type or mutant dominant negative Cx43 (T154A) (synthesized by GenScript, NJ, USA) using Lipofectamine (Invitrogen) and G418 neomycin antibiotic as selection pressure (Sigma-Aldrich).

### 4.12. qRT-PCR

RNA samples were isolated and treated as described in Section 4.7. Human and mouse Cx43, Cx26 and actin expression levels were determined by qRT-PCR using specific QuantiTect Primer Assays and miScript SYBR Green PCR Kit according to the manufacturer’s protocols (Qiagen) and analyzed according to the Δ/ΔCt method. PCRs were performed in a LightCycler 480 Instrument and LightCycler 480 Software (Roche). Primers for ZEB2 (forward: 5′-CAT GAA CCC ATT TAG TGC CA-3′, reverse: 5′-AGC AAG TCT CCC TGA AAT CC-3′); CD8 (forward: 5′-GCT CAG TCA TCA GCA ACT CG-3′, reverse: 5′-ATC ACA GGC GAA GTC CAA TC-3′); and gp100 (forward: 5′-AGC ACC TGG AAC CAC ATC TA-3′, reverse: 5′-CCA GAG GGC GTT TGT GTA GT-3′) were synthetized by IDT Integrated DNA Technologies (Fermelo, Providencia, Chile). Human Hypoxia Signaling miScript miRNA PCR Arrays (MIHS-121ZF-2; Qiagen) were used for determination of hypoxia-induced miRNAs in pooled RNA samples of Mel1, Mel2, and Ml3 melanoma cells following the manufacturer protocols. 

### 4.13. Western Blot

Cells were lysed in radioimmunoprecipitation assay (RIPA) lysis buffer supplemented with 5 mM EDTA and protease/phosphatase inhibitors cocktail (all from Thermo Fisher Scientific, Waltham, MA, USA) for 30 min on ice. Western blotting was conducted as described previously [25], loading 30 μg of total proteins per well. For protein detection HRP-conjugated anti-Cx43 (dilution 1:500; clone F-7; Santa Cruz Biotechnology), and HRP-conjugated anti-β-actin (dilution 1:10,000; clone C4; Santa Cruz Biotechnology) antibodies were used. The membranes were revealed using the SuperSignal^®^ West Pico Chemiluminescent Substrate kit (Thermo Fisher Scientific) according to manufacturer’s instructions and analyzed in an ImageQuant LAS 500 (GE Healthcare, Uppsala, Sweden). Images were analyzed using ImageJ software (National Institutes of Health). Unedited image of the western blot is shown in Figure S7.

### 4.14. Statistics

Data were analyzed using GraphPad Prism 7 (GraphPad Software Inc., San Diego, CA, USA). Statistical analyses were performed using one-way ANOVA, Tukey’s multiple comparison tests, or a two-tailed Student’s *t*-test where appropriate. Differences were considered statistically significant at *p* < 0.05.

## Figures and Tables

**Table 1:** Experimentally validated murine miR-192-5p and miR-148-3p targets.

Target ^1^	Description	Relevant Immune Role	Ref.
miR-192-5p			
ZEB2	Zinc finger E-box binding homeobox 2	Effector T cell and DC differentiation and function	[35]
miR-148a-3p			
Camk2a	Calcium/calmodulin dependent protein kinase II alpha	DC maturation and function	[38]
Kdm6b	Lysine demethylase 6B	Proinflammatory cytokine production by DCs	[39]
Rock1	Rho associated coiled-coil containing protein kinase 1	DC antigen presentation	[40]
Dnmt1	DNA methyltransferase 1	Tumor associated-DC maturation	[41]
Fcgr2b	Fc fragment of IgG receptor IIb	Tumor antigen uptake and DC activation	[42]
Epas1	Endothelial PAS domain protein 1 (HIF2A)	Regulatory T cell function	[43]

^1^ Only targets identified through strong evidence validation methods (reporter assay, western blot or qPCR). The miRNA targets were found using the miRTarBase 2020 database (http://miRTarBase.cuhk.edu.cn/).

## Supplementary Materials

The following are available online at https://www.mdpi.com/1422-0067/21/20/7567/s1. Figure S1: Cx43 expression and gap junction intercellular communication in stably transfected cells, Figure S2: Human monocyte-derived dendritic cells acquire miR-210-3p and miR-449a from hypoxic Mel3 melanoma cells, Figure S3: Correlations of ZEB2 and granzymes mRNA expression in cancer patients, Figure S4: Main antitumor roles of Cx43-GJICs in the cancer-immunity cycle, Figure S5: Proposed model for Cx43-dependent suppression of cytotoxic activity of cytotoxic T lymphocytes by hypoxic melanoma cells, Figure S6: Cytotoxic T lymphocytes acquire miR-210-3p from hypoxic B16F10 cells, Figure S7: Unedited western blot image related to Figure 1D.

## Abbreviations

BM-DC	Bone marrow-derived DC
CBX	Carbenoxolone
cGAMP	Cyclic guanosine monophosphate-adenosine monophosphate
CTL	Cytotoxic T lymphocyte
Cx	Connexin
DC	Dendritic cell
EV	Extracellular vesicles
H	Hypoxia
HGR	Hypoxia green reagent
HIF	Hypoxia induced factor
HRM	Hypoxia responsive miRNA
GJ	Gap junction
GJA1	Gap junction protein alpha 1 (Cx43)
GJB2	Gap junction protein beta 2 (Cx26)
GJIC	Gap junction-mediated intercellular communication
GZM	Granzyme
MFI	Mean fluorescence intensity
miRNA	microRNA
mo-DC	Monocyte-derived DC
N	Normoxia
NK	Natural killer
PD-1	Programmed death-1
PD-L1	PD-ligand 1
pMHC	Major histocompatibility-antigen peptide complex
shRNA	Short hairpin RNA
TAA	Tumor associated antigen
TCGA	The cancer genome atlas
TCR	T cell receptor
TME	Tumor microenvironment
