# Supplementary material for: Hypoxic Melanoma Cells Deliver microRNAs to Dendritic Cells and Cytotoxic T Lymphocytes through Connexin-43 Channels
Source: Int J Mol Sci. 2020 Oct 13;21(20):7567. doi: 10.3390/ijms21207567 (PMC7589225; doi:10.3390/ijms21207567)
Supplement: Supplementary file 1 [file ijms-21-07567-s001.pdf]

Supplementary Material

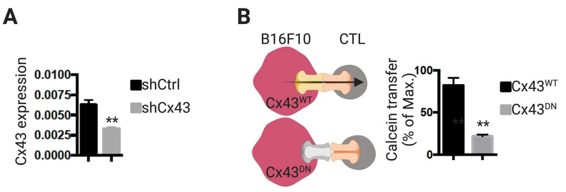

Supplementary Figure 1: Cx43 expression and gap junction intercellular communication in stably transfected cells. (**A**) Cx43 expression was determined by qRT-PCR in B16F10 melanoma cells stably transfected with plasmids expressing short hairpin RNAs against Cx43 (shCx43) or control shRNAs (shCtrl) and cultured in hypoxia (1% O_2) for 24 hours. Cx43 expression levels were obtained by the Δ/ΔCt method and normalized to actin expression. (**B**) Hypoxic (24 hours 1% O_2) B16F10 cells expressing wild type or a dominant negative Cx43 mutant (Cx43^{WT}, Cx43^{DN}, respectively), were pre-loaded with calcein-AM and co-cultured for 1 hour with Violet BMQC-pre-loaded pMEL-1 cytotoxic T lymphocytes (CTLs), at a 1:5 ratio. Calcein transfer from melanoma to CTLs was assessed by flow cytometry. The bar graph shows calcein transfer (Violet BMQC$^+$calcein$^+$ cells) as percentage of the maximum.

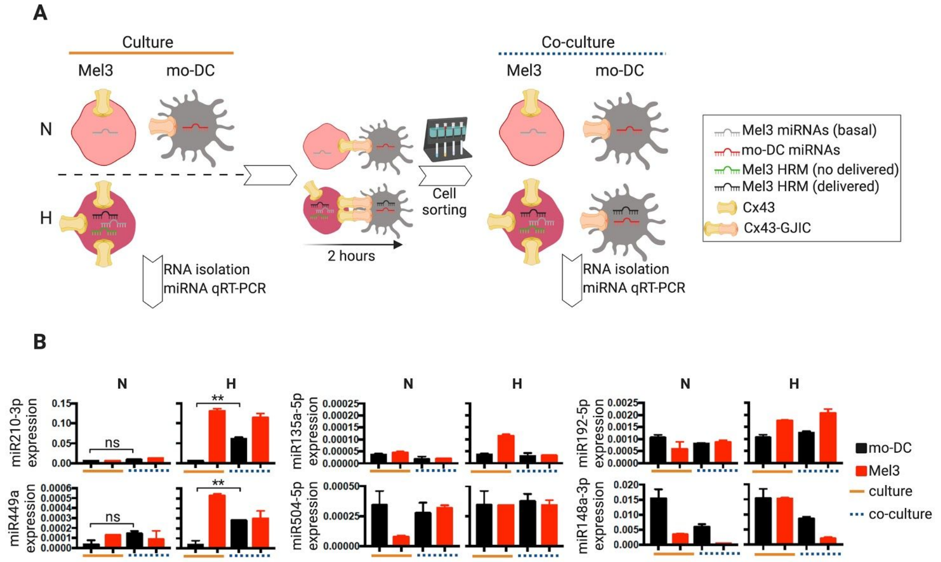

Supplementary Figure 2: Human monocyte-derived dendritic cells acquire miR210-3p and miR449a from hypoxic Mel3 melanoma cells. (**A**) Experimental scheme. Mel3 melanoma cells were cultured for 72 hours upon normoxic (21% O_2, N) or hypoxic (1% O_2, H) conditions. Then, N and H melanoma cells were co-cultured for 2 hours with monocyte-derived dendritic cells (mo-DC) upon a 1:2 cell ratio. After co-culture, mo-DC and melanoma cells were isolated using CD11c$^+$ magnetic cell sorting. Total RNA samples (including the miRNAs) were isolated from pre-co-culture (named culture, orange lines) and post-co-culture (named co-culture, blue dashed lines) cells and miRNA expression analysed by qRT-PCR. (**B**) The expression levels of miR-210-3p, -449a (left), -135a-5p, -504-5p (middle), -192-5p, and -148a-3p (right), were evaluated by qRT-PCR assays in the cells obtained as described in (A). The levels of miRNA expression were obtained by the Δ/ΔCt method and normalized to small nuclear RNA RNU6-2. ** $p < 0.01$; ns: non-significant (two-tailed Student's *t*-test).

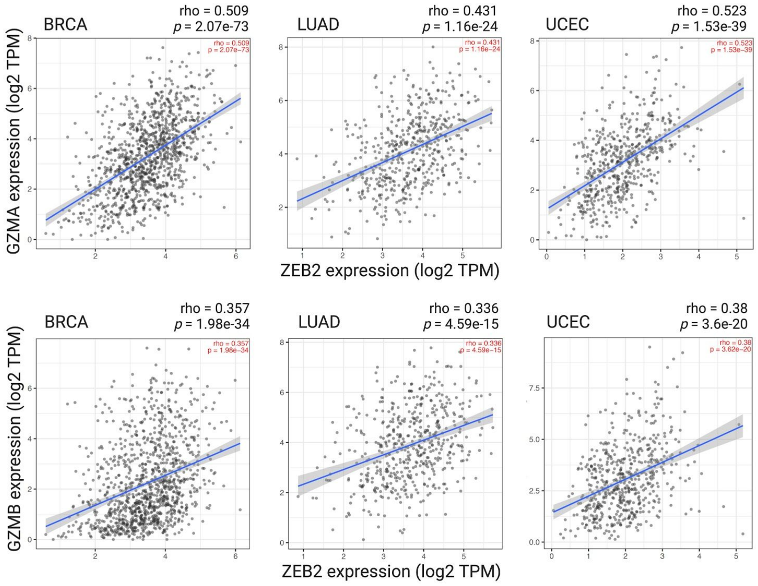

Supplementary Figure 3: Correlations of ZEB2 and granzymes mRNA expression in cancer patients. Correlations of GZMA, GZMB and ZEB2 expression levels in The Cancer Genome Atlas (TCGA) database estimated by TIMER2.0 resource (http://timer.cistrome.org/) in breast invasive carcinoma (BRCA; n = 1100 patients), lung adenocarcinoma (LUAD; n = 515 patients), and uterine corpus endometrial carcinoma (UCEC; n = 545 patients). Spearman's rho and *p* values are shown.

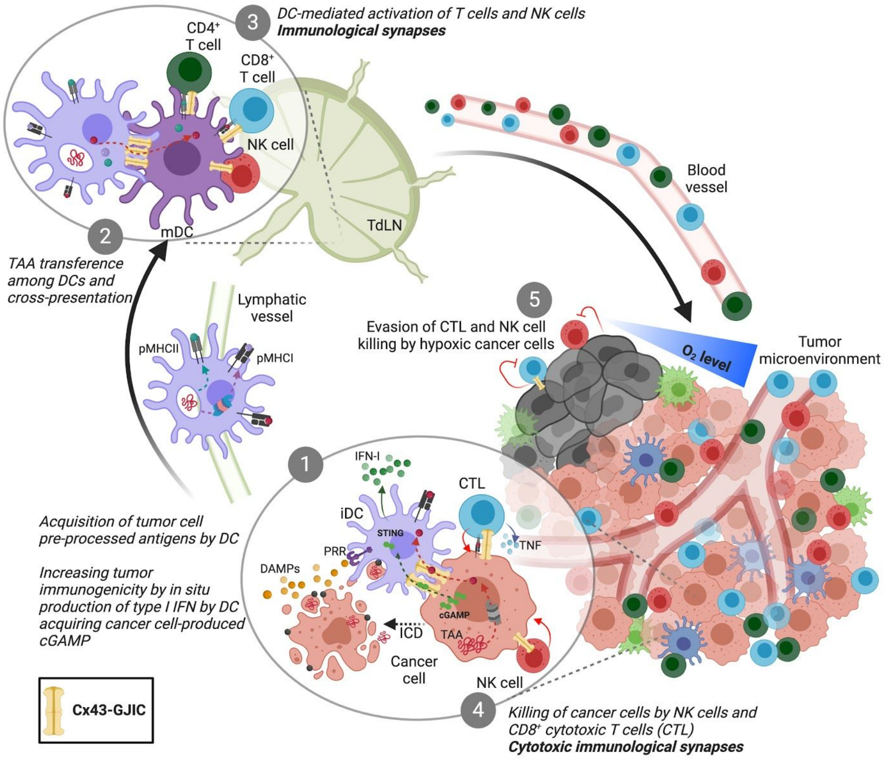

Supplementary Figure 4: Main antitumor roles of Cx43-GJICs in the cancer-immunity cycle. 1. Cx43 expression in melanoma cells allows the transfer of pre-processed tumor associated antigens (TAAs) from melanoma cells to dendritic cells (DCs), improving DC-based tumor vaccination by increasing $CD8^{+}$ T cell activation and antitumor immunity [1]. Cancer cells produce cGAMP that is transferred via Cx43-GJs to

tumor-associated DCs and macrophages, which respond by producing type I IFN *in situ*, thus, increasing tumor immunogenicity [2]. 2. Cx43-GJs participate in melanoma antigen transfer and cross-presentation between human DCs, facilitating a more effective DC-mediated T cell activation [3]. 3. Cx43-GJs accumulate at the immunological synapse formed between DCs and melanoma-specific CD4+ T cells, CD8+ T cells and natural killer (NK) cells, contributing to lymphocyte activation [4-6]. 4. Cx43-GJs accumulate at the cytotoxic immunological synapses formed between NK cells or CD8+ cytotoxic T lymphocytes (CTL) and melanoma cells, contributing to granzyme b-mediated induction of apoptosis in the target cells [5-8]. 5. The hypoxic tumor microenvironment decreases the susceptibility of melanoma cells to cytotoxic NK cell-mediated elimination via the autophagy mediated degradation of Cx43-GJ [7], and as suggested by our results, by transferring hypoxia-induced miRNAs to CTLs and repressing their cytotoxic activity. DAMPs: danger associated molecular patterns; ICD: immunogenic cell death; TdLN: tumor-draining lymph node.

References for Supplementary Figure 4:

1. Saccheri, F.; Pozzi, C.; Avogadri, F.; Barozzi, S.; Faretta, M.; Fusi, P.; Rescigno, M. Bacteria-induced gap junctions in tumors favor antigen cross-presentation and antitumor immunity. *Sci. Transl. Med.* **2010**, 2, 44ra57.
2. Schadt, L.; Sparano, C.; Schweiger, N.A.; Silina, K.; Cecconi, V.; Lucchiari, G.; Yagita, H.; Guggisberg, E.; Saba, S.; Nascakova, Z.; et al. Cancer-Cell-Intrinsic cGAS Expression Mediates Tumor Immunogenicity. *Cell Rep.* **2019**, 29, 1236–1248.
3. Mendoza-Naranjo, A.; Sáez, P.J.; Johansson, C.C.; Ramírez, M.; Mandakovic, D.; Pereda, C.; López, M.N.; Kiessling, R.; Sáez, J.C.; Salazar-Onfray, F. Functional gap junctions facilitate melanoma antigen transfer and cross-presentation between human dendritic cells. *J. Immunol.* **2007**, 178, 6949–6957.
4. Mendoza-Naranjo, A.; Bouma, G.; Pereda, C.; Ramírez, M.; Webb, K.F.; Tittarelli, A.; López, M.N.; Kalergis, A.M.; Thrasher, A.J.; Becker, D.L.; et al. Functional gap junctions accumulate at the immunological synapse and contribute to T cell activation. *J. Immunol.* **2011**, 187, 3121–3132.
5. Tittarelli, A.; Mendoza-Naranjo, A.; Farías, M.; Guerrero, I.; Ihara, F.;Wennerberg, E.; Riquelme, S.; Gleisner, A.; Kalergis, A.; Lundqvist, A.; et al. Gap junction intercellular communications regulate NK cell activation and modulate NK cytotoxic capacity. *J. Immunol.* **2014**, 192, 1313–1319.
6. Tittarelli, A.; Navarrete, M.; Gleisner, M.A.; Gebicke-Haerter, P.; Salazar-Onfray F. Connexin-Mediated Signaling at the Immunological Synapse. *Int. J. Mol. Sci*. **2020**, 21, 3736.
7. Tittarelli, A.; Janji, B.; Van Moer, K.; Noman, M.Z.; Chouaib, S. The selective degradation of synaptic connexin 43 protein by hypoxia-induced autophagy impairs natural killer cell-mediated tumor cell killing. *J. Biol. Chem.* **2015**, 290, 23670–23679.
8. Hofmann, F.; Navarrete, M.; Álvarez, J.; Guerrero, I.; Gleisner, M.A.; Tittarelli, A.; Salazar-Onfray, F. Cx43-Gap Junctions Accumulate at the Cytotoxic Immunological Synapse Enabling Cytotoxic T Lymphocyte Melanoma Cell Killing. *Int. J. Mol. Sci.* **2019**, 20, 4509

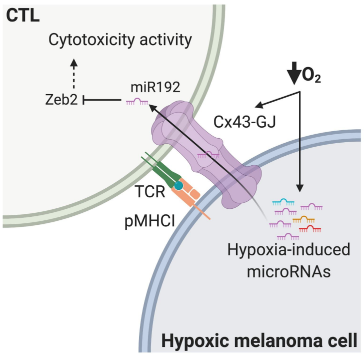

Supplementary Figure 5: Proposed model for Cx43-dependent suppression of cytotoxic activity of cytotoxic T lymphocytes by hypoxic melanoma cells. We identified that Cx43 and miR-192-5p expressions were strongly induced in hypoxic melanoma cells. This miRNA was acquired by cytotoxic T lymphocytes (CTLs) after

co-culture with hypoxic melanoma cells by a cell-contact-dependent and Cx43-mediated mechanism. Associated with the acquisition of miR-192-5p, the levels of Zeb2 (a strongly validated target of miR-192-5p) decreased in CTL after co-culture with hypoxic melanoma cells. The diminished Zeb2 expression could explain the lower cytotoxic activity of the CTLs.

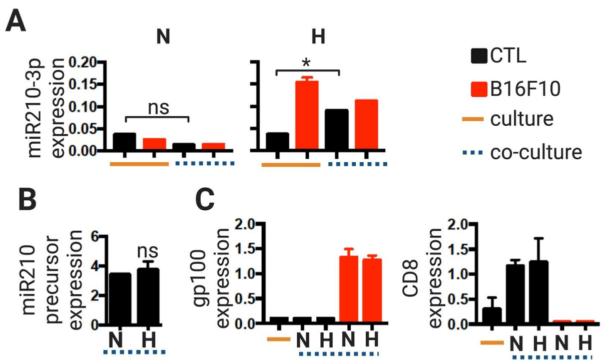

Supplementary Figure 6: Cytotoxic T lymphocytes acquire miR210-3p from hypoxic B16F10 cells. (**A**) B16F10 melanoma cells were cultured for 24 hours upon normoxic (21% O_2, N) or hypoxic (1% O_2, H) conditions. Then, N and H melanoma cells were co-cultured for 2 hours with pMEL-1 cytotoxic T lymphocytes (CTL) upon a 1:2 cell ratio. After co-culture, CTLs and melanoma cells were isolated by $CD8^+$ magnetic cell sorting. Total RNA samples (including the miRNAs) were isolated from pre-co-culture (named culture, orange lines) and post-co-culture (named co-culture, blue dashed lines) cells, and miR-210-3p (**A**), miR210 precursor (**B**), gp100 or CD8 mRNA (**C**, left and right, respectively) expression levels were analysed by qRT-PCR. Expression levels were obtained by the Δ/ΔCt method and normalized to small nuclear RNA RNU6-2 (mature and precursor miRNAs) or actin (mRNAs). * $p < 0.05$; ns: non-significant (two-tailed Student's *t*-test).

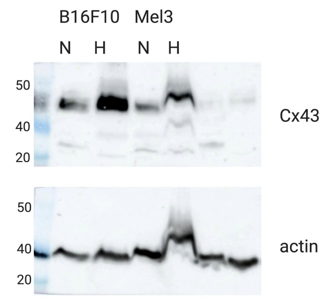

Supplementary Figure 7: Unedited western blot image related to Figure 1 D.
